# Burnout and peritraumatic distress of healthcare workers in the COVID-19 pandemic

**DOI:** 10.1186/s12889-021-11978-0

**Published:** 2021-11-12

**Authors:** Yeonhoon Jang, Myoungsoon You, Heeyoung Lee, Minjung Lee, Yeji Lee, Jin-Ok Han, Jeong Hyeon Oh

**Affiliations:** 1 Department of Public Health Science, Graduate School of Public Health, Seoul National University, 1 Gwanak-ro, Gwanak-gu, Seoul, 08826 South Korea; 2 Institute of Health and Environment, Seoul National University, Seoul, 08826 Korea; 3 Gyeonggi Public Health Institute, 172 Dolma-ro, Bundang-gu, Seongnam, Gyeonggi-do 13605 South Korea; 4 Office of Dental Education, School of Dentistry, Seoul National University, Seoul, South Korea; 5 Gyeonggi Infectious Disease Control Center, 172 Dolma-ro, Bundang-gu, Seongnam, Gyeonggi-do 13605 South Korea

**Keywords:** COVID-19, Pandemic, Healthcare workers, Mental health, Traumatic distress

## Abstract

**Background:**

To evaluate the current status of emotional exhaustion and peritraumatic distress of healthcare workers (HCWs) in the COVID-19 pandemic, and identify factors associated with their mental health status.

**Methods:**

An online survey involving 1068 of consented HCWs that included nurses, physicians, and public health officers was conducted in May 2020. Descriptive statistics and multivariate regression analyses were performed on the collected data.

**Results:**

Although no significant difference in peritraumatic distress was observed among the surveyed HCWs, the workers’ experience of emotional exhaustion varied according to work characteristics. Respondents who were female, older, living with a spouse, and/or full-time workers reported higher levels of emotional exhaustion. Public health officers and other medical personnel who did not have direct contact with confirmed patients and full-time workers had a higher level of peritraumatic distress. Forced involvement in work related to COVID-19, worry about stigma, worry about becoming infected, and perceived sufficiency of organizational support negatively predict emotional exhaustion and peritraumatic distress.

**Conclusions:**

Job-related and emotional stress of HCWs should not be neglected. Evidence-based interventions and supports are required to protect HCWs from mental illness and to promote mental health of those involved in the response to the COVID-19 pandemic.

## Background

Psychological distress of multiple individuals is intensifying due to the continuing spread of the novel coronavirus, SARS-CoV-2, the virus that causes COVID-19, and the economic downturn caused by COVID-19 [1, 2]. Accordingly, there is an emerging need for updates to policies concerning COVID-19 to prevent adverse mental health outcomes such as emotional exhaustion and traumatic distress associated with this disease [3, 4]. Healthcare workers (HCWs) in particular have been affected by the COVID-19 pandemic [5–7]. Medical personnel including physicians, nurses, and public health officers as well as those engaged in epidemiological investigation, contact tracing, management of data for individuals who have confirmed COVID-19, and those suspected of being infected have been working extensively since the early stages of the pandemic [6–8]. Infection of HCWs have been reported, some of which results in their death. As of May, 2020, a systematic review research reported that more than 150,000 infections and 1400 deaths in HCWs had been reported in worldwide [9, 10].

Some HCWs have been forced to be involved in efforts to control this infectious disease and the pandemic. Working rapidly under high-pressure and uncertain conditions, HCWs treating COVID-19 patients face challenges associated with increased workloads and high-intensity work [8]. Continuous presence of confirmed COVID-19 cases and mandatory use of personal protective equipment (PPE) in high temperature environments, together with a lack of adequate rest time, have negative impacts on physical and mental health of HCWs and contributes to physical and psychological exhaustion [9]. Previous studies that examined other infectious diseases such as severe acute respiratory syndrome (SARS) and middle east respiratory syndrome (MERS) showed that HCWs can experience serious emotional stress during the outbreak [11–14]. Moreover, outbreaks of infectious disease can increase the likelihood that HCWs will experience burnout, traumatic stress, and other mental health symptoms even after the outbreak [15, 16]. Emotional exhaustion of HCWs in the COVID-19 pandemic is particularly serious.

Earlier studies reported that female nurses and physicians experienced higher burnout rates than male counterparts [17]. Furthermore, outbreaks of infectious diseases such as SARS, MERS, and avian influenza (AI) increase the anxiety of HCWs during working hours that contributes to traumatic stress [9–11, 18]. Peritraumatic distress is emotional and physiological distress experienced during and/or immediately after a traumatic event, and is particularly related to the development and severity of posttraumatic stress disorder (PTSD) and related mental health challenges [19]. In a meta-analysis, peritraumatic distress was associated with PTSD or other psychiatric outcomes [20]. Numerous studies reported that COVID-19 has negative impacts on the mental and psychological health of HCWs and that these impacts are manifested as burnout, depression, anxiety, traumatic stress, insomnia, and even suicide [21–27].

Such psychological distress could be worsened when people around HCWs avoid them based on stigma or fear [28, 29]. Stigma, an attribute that extensively discredits an individual, involves subjective perceptions of stigmatization that can be important in predicting health-relevant adverse outcomes [30]. Stigma has negative impacts on mental health [31], and HCWs who are stigmatized reported higher stress [18], especially those who were contact with confirmed patients during the SARS pandemic [29]. Relevant research found that factors relevant to infectious diseases situations, such as shortage of PPE [32] and increased perception of worry about infection, in addition to the availability of support from their organization, can exacerbate or mitigate mental health effects for HCWs.

The lack of effective of vaccines or treatments, and the potential for a second wave of the COVID-19 pandemic in late 2020 [33] adds to the emotional burden for HCWs. In this context, the mental well-being of HCWs plays a key role during the pandemic because it is directly related to the maintenance of healthcare systems. The probability that a sustained psychological impact on HCWs can persist even after a pandemic has already been verified [15, 16]. Sustained increases in the number of confirmed patients and a constant influx of suspected cases can increase the vulnerability of HCWs to physical injuries [34], but may also worsen mental health and quality of life, which could in turn affect patient care, as well as directly increase costs at an organizational and societal level to maintain staffing levels of HCWs. Given the unpredictable nature of the COVID-19 pandemic, identifying factors that influence psychological distress of HCWs is needed to mitigate the effects of a long period of COVID-19 spread and to establish sustainable infectious disease control systems.

Several studies have examined the mental health of medical staff including nurses and physicians during the COVID-19 pandemic. However, to our knowledge, no studies have simultaneously analyzed medical staff and public health center workers. To control and deal with infectious diseases like COVID-19 properly, determining which occupations involved in responses to COVID-19 have the most serious psychological effects is important, as is identifying which factors influence the psychological wellbeing of these professionals. In this study we examined the varying psychological impacts of COVID-19 on HCWs and other professional involved in the response to COVID-19 and how to alleviate and avoid adverse outcomes associated with this pandemic.

## Methods

### Participants and procedures

Quantitative data used in this study were collected from HCWs using an online survey conducted in a specific region of South Korea, Gyeonggi-do province, between May 18 and 31, 2020. A license was obtained in order to administer the survey. During the survey period, the region recorded an average of 7.5 newly-confirmed COVID-19 cases and the range was 0-21 new patients per day.

The survey targeted HCWs at 19 hospitals, and 48 institutions engaged in disease prevention in the Gyeonggi-do province. These institutions included the provincial government, and public healthcare centers that work to provide healthcare to patients who were confirmed to have COVID-19 or those who were screened or part of epidemiological investigations. HCWs who were surveyed included: (i) Medical staff working in hospital including nurses, and physicians, and medical technicians; (ii) Field response workers such as epidemiological investigators and public healthcare center officers who were encouraged to voluntarily participate in the survey through an official document; (iii) Nurses and physicians involved in invasive work who had contact with COVID-19 patients or collected saliva or other samples, as well as those involved in diagnostic testing for patient wards or the ICU; (iv) Nurses and technicians engaged in non-invasive work such as consulting, providing education; and (v) Workers not directly providing service to confirmed COVID-19 patients such as clerical workers. Workers who performed field work such as managing COVID-19 patient data, performing screening tests, allocating beds for confirmed patients, transferring patients, and conducting field epidemiological investigations were also included in the survey.

### Measures

#### Dependent variables

Respondents completed an online self-report questionnaire that included questions that covered several areas: (1) Emotional Exhaustion and (2) Peritraumatic Distress. Emotional exhaustion of the respondents was measured with Maslach Burnout Inventory (MBI-EE) that included 9 items rated using a 7-point Likert-type scale (0 = never, 6 = every day) [35]; the Cronbach’s alpha in this study was 0.93. The Peritraumatic Distress Index (PDI) was used to measure the peritraumatic distress of HCWs. The PDI includes 13 items that assess experiences during work associated with COVID-19. The PDI measures a broad range of emotional and physical reactions to traumatic situations. The index is rated on a 5-point Likert-type scale ranging from 0 to 4 (0 = not at all, 4 = very much). Inner reliability was 0.89.

#### Independent variables

Independent variables measured included: demographic and work characteristics; worry about stigma; worry about infection; and work-related support. The demographic characteristics of participants included sex (1 = male, 2 = female), age, and presence of spouse (1 = Yes, 2 = No). We also collected job-related information, such as occupation (1 = nurse, 2 = doctors, 3 = technicians, 4 = public health officer), employment status (1 = permanent, 2 = temporary), and whether the individual had contact with COVID-19 patients (1 = yes, 2 = no).

We developed a stigma questionnaire using three items ranging from 1 (highly disagree) to 4 (highly agree). For example, ‘I worry people around me would avoid me because of my work involving COVID-19’; ‘I worry people around me would avoid my family due to my work involving COVID-19’. Worry about infection was measured with a single item question, rated on an 11-point Likert-type scale ranging from 0 to 10 (0 = not at all, 10 = very much).

Respondents were also asked to use an 11-point Likert-type scale (0 = very insufficient, 10 = very sufficient) to rate the perceived sufficiency of support from their organization while working in the COVID-19 pandemic, including safety training and education, break time, and psychological support. Finally, study participants reported their perception of the degree of coercive participation in COVID-19 work (1 = strongly disagree, 4 = strongly agree).

### Statistical analysis

We performed statistical analyses using R version 4.0.2 (R Foundation for Statistical Computing, Vienna, Austria). All descriptive statistic results of quantitative variables were reported as either the number of responses (percentage %) or mean (M) and standard deviation (SD). We used a t-test and ANOVA to identify differences in responses by demographic factors and occupational factors. To name work types, we combined two job-related variables and recategorized job-related characteristics (i.e., nurses in contact with COVID-19 patients (NC); nurses not in contact with COVID-19 patients (NNC); other medical staff that had contact with COVID-19 patients (MC); other medical staff that did not have contact with COVID-19 patients (MNC); public health officers who had no contacts with COVID-19 patients (PNC)). Multivariate linear regression analyses were performed to examine the effect of demographic factors, job-related factors associated with stigma, and work-related support to address peritraumatic distress and emotional exhaustion.

## Results

### Descriptive statistics

Descriptive statistics are shown in Table 1. In total, 1112 HCWs participated in this survey for a 74.1% response rate. Of these, data for 1068 respondents were used for the analysis. Among the respondents, 156 (14.6%) were men and 912 (85.4%) were women. The mean age was 35 years-old (M = 35.2, SD = 9.1; range = 20–69) and 54.3% of respondents had a spouse. Occupation and COVID-19 work type was combined and recategorized as: nurses who did (*n* = 512) and did not (*n* = 143) have contact with confirmed COVID-19 patients; physicians or technicians who did (*n* = 92) and did not (*n* = 60) have contact with confirmed COVID-19 patients; and public health officers not working in contact with patients with COVID-19 (*n* = 261). Among the respondents, 82.3% were employed full-time.

**Table 1:** Characteristic of demographic factors

Variable	N (%)
**Sex**	
Male	156 (14.6)
Female	912 (85.4)
**Age (Years)**	
< 30	398 (37.3)
30 ~ 39	352 (33.0)
40 ~ 49	207 (19.4)
≥50	111 (10.4)
**Spouse**	
Yes	490 (45.9)
No	559 (52.3)
**Occupation**	
Doctors, technicians	152 (14.2)
Nurse	655 (61.3)
Public health officer	261 (24.4)
**Contact with patients or suspected**	
Yes	475 (44.5)
No	593 (55.5)
**Employment**	
Full-time	879(82.3)
Part-time	189(17.7)

### Mean score of variables

ANOVA and a post-hoc test were performed for variables that included emotional exhaustion; peritraumatic distress; worry about infection; worry about stigma; and perceived degree of organizational support. Significant differences were found among work type (Table 2). Nurses who worked in contact with confirmed COVID-19 patients showed the highest average score for emotional exhaustion compared to other work types (*p* <  0.001). Emotional exhaustion of NC was higher than MC (*p* <  0.001) and MNC (p <  0.001), and PNC’s exhaustion was higher than MC (*p* <  0.001), and MNC (*p* = 0.017). Emotional exhaustion of NNC was significantly higher than that of MNC. A statistically significant difference in peritraumatic distress was identified among HCWs (*p* = 0.003). Post-hoc tests showed that the PDI score for MNC was significantly higher than NC (*p* = 0.016) and NNC (*p* = 0.017), and PNC (p = 0.003). Worry about infection was significantly higher for nurses than for MC (*p* <  0.001). In terms of the perception of pressures associated with COVID-19 work participation, PNC were more likely to perceive the participation in COVID-19 work as being coercive relative to other workers (M = 3.7, *p* <  0.001). Differences between occupational characteristics were significant for stigma, and post-test results showed that the worry about stigma for MC NC was significantly lower than that for NC (*p* = 0.001) and NMC (*p* = 0.03). Safety training and education opportunities were considered to be the least sufficient by PNC, whereas MNCs were most likely to indicate that the opportunities were sufficient. Post-test results showed that PNC perceived that the amount of safety training and education was less than that by other professions (*p* <  0.001). MNCs also indicated that they had more education than NNC (*p* = 0.009). PNC showed the lowest degree of sufficient rest time compared to other professions. Post-test results showed that PNC perceived that they had less rest time than MC (*p* <  0.001) and MNC (*p* <  0.001) and that NCs perceived having less rest time than MC (*p* = 0.02) and MNCs (*p* = 0.005). Finally, the sufficiency of psychological support was the highest for MNC, whereas public health officials had the lowest level. In the post-test, NC and PNC indicated that availability of psychological support was less than that of MC (*p* = 0.002) and MNC (*p* = 0.001).

**Table 2:** Descriptive statistics of variables by job characteristics

Variable	Total	NC (***N*** = 512)	NNC (***N*** = 143)	MC (***N*** = 92)	MNC (***N*** = 60)	PNC (***N*** = 261)	***p***-value^**1**^
**Emotional Exhaustion**	30.8 ± 12.5	32.5 ± 11.4	30.8 ± 11.5	23.6 ± 13.6	25.8 ± 13.2	31.2 ± 13.5	< 0.001
**Peritraumatic Distress**	19.1 ± 9.1	19.2 ± 8.9	19.8 ± 8.7	16.0 ± 10.0	17.5 ± 8.8	20.0 ± 9.5	0.003
**Worry for infection**	6.6 ± 2.3	6.8 ± 2.2	6.8 ± 2.1	6.0 ± 2.7	6.1 ± 2.6	6.5 ± 2.3	0.002
**Perception of Coercive Participation in the COVID-19 Work**	3.1 ± 1.2	2.9 ± 1.2	2.7 ± 1.2	2.8 ± 1.4	2.7 ± 1.1	3.7 ± 1.1	< 0.001
**Worry for stigma**	2.4 ± 1	2.6 ± 0.9	2.3 ± 0.9	2.3 ± 1.0	2.1 ± 0.9	2.2 ± 1.0	< 0.001
**Training, Education for Safety**	5.8 ± 2.4	5.9 ± 2.2	5.9 ± 2.1	6.2 ± 2.5	7.1 ± 2.2	4.9 ± 2.5	< 0.001
**Breaktime**	4.8 ± 2.8	4.8 ± 2.6	4.9 ± 2.5	5.7 ± 2.9	6.0 ± 2.7	4.3 ± 2.9	< 0.001
**Psychological support**	3.5 ± 2.5	3.3 ± 2.5	3.9 ± 2.4	4.2 ± 2.8	4.4 ± 2.8	3.2 ± 2.4	< 0.001

(Data are presented in the form of average ± SD)

^1^*P*-values of variables among work type (i.e., NC, NNC, MC, MNC, PNC)

To determine whether these differences were clinically meaningful, the prevalence of high scores was calculated (Table 3). The prevalence of indicators of peritraumatic distress during the COVID-19 pandemic was higher for NNC, however, no statistical significance was observed among work characteristics (*χ*^2^ = 6.28, *p* = 0.179). The prevalence of emotional exhaustion was highest for NC (70.7%), followed by NNC (66.4%) and PNC (60.2%); these differences were statistically significant (*χ*2 = 43.33, p = 0.179).

**Table 3:** Prevalence of adverse outcomes in the COVID-19 healthcare workers

	NC (***N*** = 512)	NNC (N = 143)	MC (N = 92)	MNC (N = 60)	PNC (N = 261)	*p*-value
Peritraumatic Distress (≥23)	177 (34.6%)	55 (38.5%)	25 (27.2%)	14 (23.3%)	89 (34.1%)	0.179
Emotional Exhaustion (≥27)	362 (70.7%)	95 (66.4%)	38 (41.3%)	26 (43.3%)	157 (60.2%)	< 0.001

### Factors that influence emotional exhaustion and peritraumatic distress of HCWs in the COVID-19 pandemic

Multivariate linear regression was performed to identify factors that affect emotional exhaustion and peritraumatic distress of healthcare workers during the COVID-19 pandemic (Table 4). Female, full-time healthcare providers had higher rates of emotional exhaustion, whereas respondents who were younger, and who those who had a spouse had lower emotional exhaustion. COVID-19 work participation pressure (β = 1.58, *p* <  0.001), worry about stigma (β = 1.93, *p* <  0.001), and worry about infection (β = 0.65, *p* <  0.01) were positive and significant predictors, whereas perception of breaktime sufficiency (β = − 0.54, *p* <  0.001), and psychological support (β = − 0.66, p <  0.001) were significantly negative predictors of work-related emotional exhaustion of HCWs. These factors accounted for 31.4% of the variance in emotional exhaustion (F [14, 1053] = 35.95, adjusted R^2^ = 0.314, *p* < 0.001).

**Table 4:** Multivariable regression of peritraumatic distress

	Work-related Burnout (Exhaustion)			Peritraumatic Distress		
*Predictors*	*Estimates*	95% CI	*p-value*	*Estimates*	95% CI	*p-value*
Constants	21.02	16.08, 25.97	< 0.001	3.81	0.09, 7.54	0.045
Sex [ref: male]	4.64	2.65, 6.62	< 0.001	1.49	− 0.01, 2.98	0.051
Age	−0.09	− 0.19, − 0.00	0.046	0.01	− 0.06, 0.08	0.702
Presence of spouse [ref: no]	−3.47	−5.06, −1.87	< 0.001	− 1.15	−2.36, 0.05	0.06
Nurse (No contact) [ref: Nurse (Contact)]	0	−1.95, 1.95	1	1.47	−0.00, 2.94	0.05
Other medical staffs (contact)	−2.07	−4.66, 0.53	0.119	0.2	−1.75, 2.16	0.837
Other medical staffs (No contact)	1.5	−1.56, 4.55	0.336	2.43	0.13, 4.73	0.039
Public health officers (No contact)	0.42	−1.41, 2.24	0.654	2.09	0.72, 3.46	0.003
Employment [ref: part-time]	3.79	1.94, 5.64	< 0.001	1.94	0.55, 3.34	0.006
Coercive participation	1.58	1.02, 2.14	< 0.001	0.56	0.13, 0.98	0.01
Worry for stigma	1.93	1.22, 2.65	< 0.001	2.96	2.42, 3.50	< 0.001
Worry for infection	0.65	0.36, 0.95	< 0.001	0.89	0.66, 1.11	< 0.001
Training and education for safety	−0.23	−0.54, 0.08	0.144	−0.28	−0.51, −0.04	0.019
Breaktime	−0.54	− 0.83, − 0.25	< 0.001	−0.24	− 0.47, − 0.02	0.031
Psychological support	− 0.66	−0.99, − 0.33	< 0.001	−0.08	− 0.33, 0.16	0.514
Observations	1068					
R^2^ / R^2^ adjusted	0.323 / 0.314			0.278 / 0.268		

For peritraumatic distress, MNC, PNC, and full-time HCWs showed higher PDI scores compared to other work types. Perceived COVID-19 work participation pressure (β = 0.56, *p* < 0.01), worry about stigma (β = 2.96, *p* < 0.001), and worry about infection (β = 0.89, *p* < 0.001) had a positive effect on peritraumatic distress of the respondents. Peritraumatic distress was negatively associated with perceived sufficiency of safety training and education (β = − 0.28, *p* = 0.02), and amount of breaktime (β = − 0.24, *p* = 0.03). These factors accounted for 26.8% of the variance in peritraumatic distress (F [14, 1053] = 28.97, adjusted R^2^ = 0.268, *p* < 0.001).

## Discussion

Our study reported psychological distress of HCWs including nurses, physicians, and public health officers. We found that emotional exhaustion of HCWs is highly serious, as evidenced by an average score (30.8) that was higher than the prevalence cut-off point (27). The prevalence of emotional exhaustion of this study was 63%, which was worse than that for studies concerning SARS or MERS [15, 36]. In particular, the prevalence of burnout among nurses was the highest of the different work types examined. This result is similar to that reported in other related COVID-19 studies [37]. The higher prevalence of emotional exhaustion could be attributed to long-term COVID-19 work participation, having to work in a risky workplace, and continuous presence of COVID-19 related tasks [15, 17]. The traumatic distress of staff working in public health centers was worse than that for any other type of HCW. These results (Table 4) indicated that more attention to mental health wellbeing of nurses caring for COVID-19 patients is needed [22]. There was no significant difference in the prevalence of PDI among work characteristics, but the mean PDI score for public health officers was statistically higher than that for respondents engaged in other types of work. Furthermore, although the mean score for emotional exhaustion for NC was the highest of the work types examined, the scores for PNC were as high as the exhaustion scores for nurses. This result implies that first-line medical workers do not always have higher psychological distress than other workers, and highlights that supports such as provision of rest time, safety training and education, and psychological support are needed for all types of HCWs, regardless of their job type and obligations [25]. Based on previous reports concerning the relationship between exhaustion and psychological distress [38, 39], future research should consider the importance of psychological wellbeing of HCWs.

The results of multiple regression analyses showed that full-time employment and the perception that COVID-19 work participation was compulsive were negatively associated with psychological distress. This result could reflect the conflicting obligations of duty to care for COVID-19 patients on the one hand and the drive for self-preservation on the other. Full-time HCWs are exposed to higher risks from COVID-19 compared to part-time workers simply based on the number of hours worked. Due to their full-time status, such HCWs are more likely to be placed on response teams, which can enhance feelings of stress and perception that participation in the work is mandatory. As an infectious disease outbreak can result in decreased willingness to work [40–44], compulsory work may exacerbate negative impacts on psychological well-being in that conflicts between work obligations and worry about infection can affect mental wellbeing.

Difficulties in the workplace including insufficient break time, concern for safety, and long-term workload can also contribute to psychological distress of HCWs in the COVID-19 pandemic. Previous research found that there is indeed an association between physical health problems and workplace environmental stressors [23, 32]. Contact with confirmed COVID-19 patients or those suspected of having COVID-19, the possibility of being infected, and stigma could worsen the psychological wellbeing of HCWs [24].

The results of the present study were consistent with previous studies and showed that stigma influenced psychological health or mental illness [31, 45]. Stigmatization of HCWs affects their psychological and physical health [18, 31, 45, 46]. Particularly during outbreaks of infectious diseases, HCWs who had contact with confirmed patients felt more stigma compared to other workers. Thus, preventing stigma of HCWs is an important issue that should be addressed during catastrophic situations including pandemics [47–49]. Hospital administrators and policymakers should take appropriate actions to ensure that HCWs do not suffer from pandemic-related stigma and minimize negative effects from stigma that may occur [50].

Psychological distress of HCWs could persist for years after the outbreak, and this sustained psychological distress would be expected to have adverse effects on the physical health of these workers [15, 16, 51]. Thus, development of evidence-based interventions is needed to prevent adverse mental health problems among HCWs. The current study found that psychological supports could mitigate emotional exhaustion of HCWs who are treating patients with COVID-19, which is a similar to that reported in an earlier study [48]. Breaktime, safety training and education, and psychological support all improve mental health of HCWs [21, 52]. In this context, individual and organizational interventions need to be initiated for HCWs. Strategies such as mindfulness practices and leveraging of positive psychology resources that are readily available to individual HCWs could help them manage their mental well-being [10, 53, 54]. Another important factor that might minimize HCW burnout and traumatic stress are altruistic behaviors, which are negatively related to traumatic stress [55]. Intervention toward HCWs, such as spiritual programs, might be considered as well, which could decrease emotional exhaustion and psychological impairment [56, 57].

Organizations including hospitals, clinics, and public health institutions need to provide sufficient training and exercises to provide psychological support for employees that would mitigate the negative impact of infectious disease outbreaks on mental health [58], and guarantee sufficient rest time or flexible working hours [59]. Routine support from colleagues and supervisors enhances the perception by HCWs that they are being protected [34, 60]. Mobile health tools [61], telephone helplines [21], or digital learning packages [62] are other approaches to reduce and manage their mental illnesses associated with working during an infectious disease outbreak.

In conjunction with organizational measures, support from government, policymakers would be important as well. For instance, it could be useful to implement psychological support resources within the framework of a mandatory occupational health surveillance program, such as providing HCWs with adequate information, enhancing with psychological support of HCWs along with mental health monitoring to deal with their psychological distress. Conducting health surveillance programs with the intervention of occupational health professionals in the hospital setting could help managing both physical and mental health of HCWs [27, 63].

The strength of this study is that we surveyed both medical staff and public health officers who, unlike nurses or physicians, are typically not considered in studies of the psychological impacts of disease outbreaks. In South Korea, there is a tendency to recognizes HCWs as only including nurses and physicians. Our examination of the mental health status of public health workers in the present study could be helpful for the lay public to recognize the extent of the effects on these workers by disease outbreaks. In this study we estimated the traumatic distress of HCWs using PDI. Although most of the relevant studies use PTSD scales to estimate traumatic distress, consideration of peritraumatic distress could reveal traumatic distress during or right after the disease outbreak.

This study does have several limitations. First, there was a bias in the type of survey respondent. More than half of the participants were nurses and few physicians responded to the survey. Furthermore, only those HCWs living in Gyeonggi-do were included in the survey. Second, the variables for an objective index of work, for instance, number of hours spent working with patients with COVID-19, was not determined. We were only able to identify associations between adverse outcomes and perception and respondents’ perceptions. Finally, since this was a cross-sectional study, the significant association between psychological distress and organizational support (i.e., training, education, break time, psychological support) may not imply a causal relationship. Due to the characteristics of the PDI, we could only examine the short-term effect of COVID-19 situation on mental wellbeing of HCWs. As such, follow-up research is needed to identify long-term negative impacts of disease outbreaks on mental health aspects of HCWs such as PTSD.

## Conclusion

Our study highlights that HCWs working with patients with COVID-19 are emotionally exhausted not only by affective psychological factors (e.g., worry about stigma) but also by increased work demands during the outbreak. Regardless of occupation and work characteristic, the distress level among HCWs must be managed in a timely manner. Highly challenging working conditions of HCWs involved in COVID-19 responses could increase the risk of mental health problems. Intense workloads contribute to exhaustion of HCWs that can threaten their mental wellbeing. Thus, continuous monitoring to manage the mental health of HCWs should be implemented. Efforts devoted to early detection and prevention of mental health problems of HCWs should be put into place. Early evidence-based interventions are needed not only to maintain mental health of HCWs but also to prevent negative consequences of mental health impacts on HCWs for organizations and the overall society.

## Data Availability

There is no public access to all data generated or analyzed during this study to preserve the privacy of the identities of the individuals. The dataset that supports the conclusions is available to the corresponding author upon request.

## Abbreviations

COVID-19: Coronavirus disease 2019
HCW: Healthcare Workers
NC: nurses in contact with COVID-19 patients
NNC: Nurses not in contact with COVID-19 patients
MC: other medical staff that had contact with COVID-19 patients
MNC: Other medical staff that did not have contact with COVID-19 patients
PNC: Public health officers who had no contacts with COVID-19 patients
PDI: Peritraumatic distress Index
M: Mean
SD: Standard deviation
